# Real-world apixaban concentration in Korean patients with atrial fibrillation

**DOI:** 10.1007/s44313-025-00089-z

**Published:** 2025-07-07

**Authors:** Sun Hack Lee, Mijin Kim, Min Sun Kim, Jeongcheon Choe, Jinhee Ahn, Hyewon Lee, Junghyun Choi, Han Cheol Lee, Hyerim Kim, Kwang Soo Cha

**Affiliations:** 1 Department of Cardiology and Medical Research Institute, Pusan National University Hospital, Pusan National University School of Medicine, 179, Gudeok-Ro, Seo-Gu, Busan, 49241 Korea; 2 Department of Laboratory Medicine and Medical Research Institute, Pusan National University Hospital, Pusan National University School of Medicine, 179, Gudeok-Ro, Seo-Gu, Busan, 49241 Korea

**Keywords:** Apixaban, Atrial Fibrillation, Risk Assessment, Factor Xa, Bleeding

## Abstract

**Purpose:**

Apixaban is recommended for patients with atrial fibrillation. Although routine monitoring of plasma concentrations is not typically advised, factors such as ethnicity, sex, and comorbidities can influence these levels. Our study analyzed the plasma apixaban concentrations (PAC) in patients to explore whether these levels, along with underlying conditions, offer enhanced insights for risk stratification.

**Methods:**

This study analyzed 49 patients with atrial fibrillation who had been taking apixaban for over a month, examined factor Xa levels within 6 h post-administration, and correlated PAC with clinical characteristics such as age, body weight, estimated glomerular filtration rate (eGFR), presence of heart failure, and bleeding events.

**Results:**

The mean plasma concentration of apixaban in all patients was 160.3 ± 77.5 ng/mL. Those taking apixaban 5 mg twice daily had higher plasma concentrations than those taking 2.5 mg twice daily (191.2 ± 75.3 ng/mL vs. 137.2 ± 72.0 ng/mL, *p* = 0.014). Among the patients receiving a reduced dose, renal function and heart failure were significantly associated with plasma concentrations. No factors were associated with the plasma concentrations in patients receiving the standard dose. Notably, reduced-dose patients with heart failure had plasma concentrations comparable to those of individuals receiving the standard dose and exhibited a higher incidence of bleeding than the other groups.

**Conclusions:**

PAC measurement revealed that apixaban dosages, classified based on age, body weight, and eGFR, were generally effective. Nonetheless, heart failure may increase plasma levels and correlate with an increased bleeding risk in Korean patients on reduced doses. Therefore, tailoring apixaban prescriptions to account for heart failure and other comorbidities may enhance treatment efficacy.

**Supplementary Information:**

The online version contains supplementary material available at 10.1007/s44313-025-00089-z.

## Introduction

Anticoagulation therapy with apixaban is recommended for patients with atrial fibrillation (AF) [1]. Apixaban has demonstrated a similar stroke prevention rate with a lower incidence of bleeding than warfarin, making it the first-line agent for anticoagulation therapy [2]. While warfarin requires dose adjustment based on the prothrombin time (PT) at intervals, routine monitoring of apixaban anticoagulation activity, primarily determined by plasma levels, is generally not recommended. This is because an appropriate plasma apixaban concentration (PAC) can be achieved by adjusting the dose according to age, body weight, and glomerular filtration rate (GFR). The standard dose of apixaban is 5.0 mg twice daily. However, a dose reduction to 2.5 mg twice daily is recommended for patients meeting at least two of the following criteria: age ≥ 80 years, body weight ≤ 60 kg, or serum creatinine ≥ 1.5 mg/dL [3].

However, PAC varies not only with age, body weight, and GFR but also with other factors, including race, sex, and comorbidities [4–6]. Owing to this variation in PAC, the 2024 European Society of Cardiology (ESC) guidelines acknowledge that measuring plasma concentrations can be helpful in certain situations. These include patients with bleeding complications or thromboembolic events (e.g., ischemic stroke) despite receiving apixaban therapy [7]. Therefore, the PAC in real-world clinical practice may vary and affect risk stratification.

This study aimed to analyze PAC using residual plasma samples obtained after routine clinical blood testing in Korean patients receiving apixaban therapy. Furthermore, we sought to evaluate whether baseline characteristics and comorbidities could provide additional information for risk stratification.

## Materials and methods

### Study population

The study population consisted of patients with AF who were treated with apixaban and underwent factor Xa analysis between December 2020 and November 2021. Patients with newly diagnosed paroxysmal, persistent, long-standing persistent, and permanent patterns of AF and those taking apixaban for at least a month were included [1]. Blood tests and electrocardiography were performed routinely or as required. The remaining samples were selected randomly for use in this study. The study protocol was approved by the Pusan National University Review Board (2303–001-124) and conducted in accordance with the Declaration of Helsinki.

### Sample collection

Whole blood samples were obtained via venipuncture from patients receiving apixaban. Samples were placed in 3.2% sodium citrate (Becton Biosciences, Franklin Lakes, NJ, USA). After centrifugation at 3,000 g for 10 min at room temperature, platelet-poor plasma (PPP) was extracted from the supernatant fraction and stored at −80℃ for future use. Frozen PPP aliquots were immediately tested after being thawed to 37 °C. Samples were analyzed within a storage period of no more than 4 weeks.

### Routine coagulation test

PPP coagulation testing was performed using an automatic coagulation analyzer CS-5100 (Sysmex Corporation, Kobe, Japan). PT was measured using THROMBOREL-S (Siemens Healthcare Diagnostics, Marburg, Germany) reagent, and activated partial thromboplastin time (APTT) was assessed using ACTIN FSL (Siemens), with reference values of 10.5–13.2 s and 20.0–33.0 s, respectively. PT and aPTT were measured for all samples within 4 h of blood collection and were re-measured when the apixaban concentration was measured. Apixaban concentration was measured only in thawed samples for which PT and aPTT values deviated less than 10% from baseline, indicating sample stability.

### Apixaban concentration measurement

PAC was measured using the chromogenic anti-factor Xa test. A CS-5100 automatic hemostasis analyzer was used with the BIOPHEN Heparin LRT reagent (Hyphen BioMed, Neuville-sur-Oise, France). The control substances used were 200 ng/mL and 400 ng/mL BIOPHEN apixaban (Hyphen BioMed).

### Clinical data and outcomes

All patients underwent clinical follow-ups for 3–6 months for treatment. Clinical data and outcomes were collected through a review of medical records up to November 2022. Laboratory data, medical history, and current medications, including antiplatelet agents, were collected. The causes of bleeding and death were determined from medical records. The primary outcome measure was the PAC. Expected PAC values were obtained from the ESC guidelines [3]. The secondary outcome was a composite of major International Society on Thrombosis and Haemostasis (ISTH) bleeding and clinically relevant non-major bleeding (CRNMB) [8].

### Statistical analysis

Continuous data are shown as mean ± standard deviation, and categorical variables are displayed as numbers and percentages. Group comparisons were performed using the χ^2^ or Fisher exact test for categorical variables and Student’s t-test or the Mann–Whitney U test for continuous variables as appropriate. Logistic Regression was used to identify factors significantly associated with the study outcomes. Clinically relevant covariates (*p* < 0.05) were used for univariate analysis. All statistical analyses were performed using R version 4.2.2 (R Foundation for Statistical Computing).

## Results

### Study population

A total of 63 patients were screened. Of these, 14 were excluded because of discontinuation or modification of medication, lack of follow-up during outpatient visits, or blood sampling after visiting the emergency department for bleeding events. A final analysis was conducted on 49 patients, with a mean age of 74.2 ± 7.7 years. Among the included patients, 28 were on a reduced dose of 2.5 mg twice daily, and 21 were on a standard dose of 5.0 mg twice daily. Significant differences were also observed in age (77.4 ± 6.7 vs. 70.0 ± 6.9, *p* = 0.001), body weight (58.6 ± 10.5 vs. 70.2 ± 9.0, *p* < 0.001), serum creatinine (1.5 ± 1.2 vs. 1.0 ± 1.2, *p* = 0.038), and creatinine clearance (43.7 ± 22.5 vs. 67.7 ± 17.2, *p* < 0.001) between the two groups (Table 1).

**Table 1:** Baseline characteristics and apixaban concentration

Daily dose	Total (*N* = 49)	2.5 mg twice daily (*N* = 28)	5.0 mg twice daily (*N* = 21)	*p*-value
Age (year)	74.2 ± 7.7	77.4 ± 6.7	70.0 ± 6.9	0.001
Male	27 (55.1%)	15 (53.6%)	12 (57.1%)	1.000
Height (cm)	161.4 ± 8.2	159.5 ± 7.9	164.0 ± 8.0	0.056
Weight (kg)	63.5 ± 11.4	58.6 ± 10.5	70.2 ± 9.0	0.000
Hypertension	22 (44.9%)	13 (46.4%)	9 (42.9%)	1.000
Diabetes mellitus	18 (36.7%)	8 (28.6%)	10 (47.6%)	0.285
Dyslipidemia	8 (16.3%)	2 (7.1%)	6 (28.6%)	0.106
Liver disease^a^	9 (18.4%)	7 (25.0%)	2 (9.5%)	0.312
Lung disease^b^	14 (28.6%)	10 (35.7%)	4 (19.0%)	0.338
Chronic kidney disease^c^	23 (46.9%)	15 (53.6%)	8 (38.1%)	0.432
Creatinine (mg/dL)	1.3 ± 0.9	1.5 ± 1.2	1.0 ± 0.2	0.038
Creatinine clearance (mL/min)	54.0 ± 23.5	43.7 ± 22.5	67.7 ± 17.2	< 0.001
Heart failure	22 (44.9%)	13 (46.4%)	9 (42.9%)	1.000
Ischemic heart disease	8 (16.3%)	5 (17.9%)	3 (14.3%)	1.000
Stroke	9 (18.4%)	6 (21.4%)	3 (14.3%)	0.790
Malignancy	9 (18.8%)	8 (29.6%)	1 (4.8%)	0.069
Paroxysmal atrial fibrillation	6 (12.2%)	3 (10.7%)	3 (14.3%)	1.000
CHA_2_DS_2_-VAS score	3.4 ± 1.5	3.8 ± 1.4	3.0 ± 1.5	0.080
Aspirin	4 (8.2%)	2 (7.1%)	2 (9.5%)	1.000
Clopidogrel	2 (4.1%)	2 (7.1%)	0 (0.0%)	0.602
Apixaban concentration (ng/mL)	160.3 ± 77.5	137.2 ± 72.0	191.2 ± 75.3	0.014

^a^Liver diseases included cirrhosis and fatty liver

^b^Lung diseases included chronic obstructive lung disease, asthma, and pulmonary tuberculosis

^c^Chronic kidney disease was defined as an estimated glomerular filtration rate of < 60 mL/min/1.73 m^2^

### Apixaban plasma concentration

The mean PAC in all patients was 160.3 ± 77.5 ng/mL. The mean PAC was significantly different between the reduced-dose group (137.2 ± 72.0 ng/mL) and the standard-dose group (191.2 ± 75.3 ng/mL) (*p* = 0.014). The patients with appropriate PAC range were 24/28 (85.7%) and 18/21 (85.7%) in the 2.5 mg twice daily and 5 mg twice daily regimens, respectively.

**Fig. 1:**
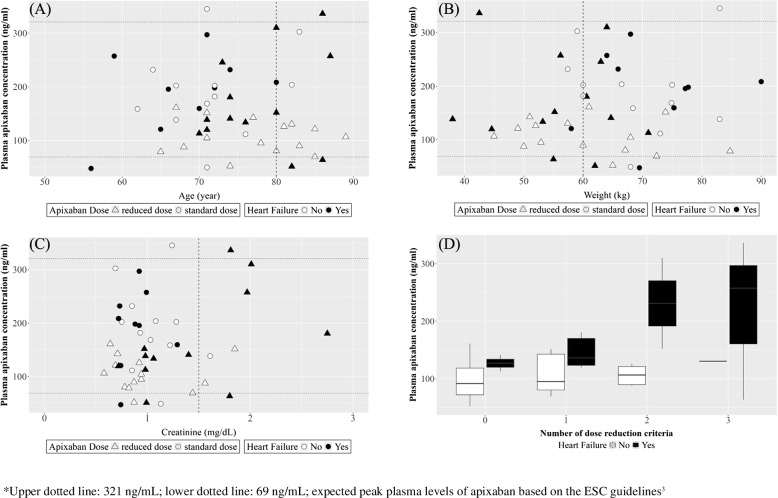
Plasma apixaban concentrations in the study population. * Upper dotted line: 321 ng/mL; lower dotted line: 69 ng/mL; expected peak plasma levels of apixaban based on the ESC guidelines [3].

The PAC distribution was examined according to the factors influencing dose determination (Fig. 1A–C). When analyzed by age, 16 patients (32.7%) were aged ≥ 80 years, with three (18.8%) receiving the standard dose. The plasma concentration for patients aged ≥ 80 years was 163.1 ± 94.8 ng/mL, compared with 159.0 ± 69.3 ng/mL for those aged < 80 years. However, no significant difference was observed between the two groups (*p* = 0.862). When stratified by body weight, 20 patients (40.8%) had a body weight ≤ 60 kg, with mean plasma concentrations of 157.1 ± 73.6 ng/mL for those with body weight ≤ 60 kg and 162.6 ± 81.3 ng/mL for those with body weight > 60 kg (*p* = 0.809). Ten patients (20.4%) had serum creatinine levels ≥ 1.5 mg/dL, with a mean plasma concentration of 190.3 ± 93.0 ng/mL, compared with 152.7 ± 72.4 ng/mL in patients with serum creatinine levels < 1.5 mg/dL, although this difference was not statistically significant (*p* = 0.173). Eleven patients (39.3%) met two or more of these criteria, with a mean plasma concentration of 162.0 ± 94.4 ng/mL compared with 121.2 ± 49.9 ng/mL in the remaining 17 patients (60.7%) (*p* = 0.498).

### Dose-specific factors affecting apixaban concentrations

To identify factors influencing PAC, linear regression analyses were conducted separately for patients receiving reduced and standard doses. In the reduced-dose group, renal function significantly affected the PAC, with serum creatinine levels and creatinine clearance showing statistical significance. In addition, heart failure (HF) was a significant risk factor (*p* = 0.012). Notably, in the standard-dose group, no significant factors were identified by linear regression analysis (Table 2). Multivariate regression analysis was performed using age ≥ 80 years, body weight ≤ 60 kg, and serum creatinine ≥ 1.5 mg/dL as criteria, with HF added to the model. The inclusion of HF improved the adjusted *R*^2^ values in the reduced-dose group (adjusted *R*^2^, 0.246 vs. 0.334) (Tables 3 and 4).

**Table 2:** Linear regression (univariate analysis) for plasma apixaban concentration

	Apixaban 2.5 mg twice daily				Apixaban 5.0 mg twice daily			
Variable	Estimated (β)	Standard error	t-value	*p*-value	Estimated (β)	Standard error	t-value	*p*-value
Age (year)	1.489	2.079	0.716	0.480	3.476	2.367	1.469	0.158
Male	−3.712	27.803	−0.134	0.895	−34.080	33.157	−1.028	0.317
Height (cm)	0.402	1.787	0.225	0.824	−0.320	2.151	−0.149	0.883
Weight (kg)	−1.479	1.309	−1.130	0.269	−0.020	1.911	−0.010	0.992
Hypertension	−15.998	27.635	−0.579	0.568	54.901	31.652	1.735	0.099
Diabetes mellitus	40.460	29.661	1.364	0.184	36.012	32.728	1.100	0.285
Dyslipidemia	66.556	52.252	1.274	0.214	2.310	37.314	0.062	0.951
Liver disease^a^	−25.566	31.638	−0.808	0.426	−42.676	56.590	−0.754	0.460
Lung disease^b^	39.994	27.865	1.435	0.163	−60.396	40.634	−1.486	0.154
Chronic kidney disease^c^	60.283	25.174	2.395	0.024	1.873	34.712	0.054	0.958
Creatinine	26.113	10.793	2.420	0.023	−23.582	70.940	−0.332	0.743
Creatinine clearance	−1.612	0.544	−2.965	0.006	−0.892	0.984	−0.907	0.376
Heart failure	66.131	24.603	2.688	0.012	−0.628	34.066	−0.018	0.985
Ischemic heart disease	45.091	35.120	1.284	0.210	27.720	47.755	0.580	0.568
Stroke	48.453	32.441	1.494	0.147	13.895	48.071	0.289	0.776
Malignancy	−30.288	30.747	−0.985	0.334	7.385	79.144	0.093	0.927
Paroxysmal atrial fibrillation	−26.267	44.549	−0.590	0.561	−87.920	43.751	−2.010	0.059
CHA_2_DS_2_-VAS score	14.045	9.482	1.481	0.151	20.575	10.647	1.932	0.068
Aspirin	48.292	53.019	0.911	0.371	28.774	57.050	0.504	0.620
Clopidogrel	52.610	52.861	0.995	0.329	Clopidogrel was not used in these patients			

^a^Liver diseases included cirrhosis and fatty liver

^b^Lung diseases included chronic obstructive lung disease, asthma, and pulmonary tuberculosis

^c^Chronic kidney disease was defined as an estimated glomerular filtration rate of < 60 mL/min/1.73 m^2^

**Table 3:** Linear regression (multivariate analysis) for plasma apixaban concentration in patients with reduced dose

		Estimated (β)	Standard error	95% CI	t-value	*p*-value	Adjusted *R*^2^	*p*-value
Model 1	Age ≥ 80 years	5.147	24.968	−46.385 to 56.680	0.206	0.838	0.246	0.020
	Body weight ≤ 60 kg	2.553	24.779	−48.588 to 53.694	0.103	0.919		
	Creatinine ≥ 1.5 mg/dL	85.960	25.508	33.314 to 138.606	3.370	0.003		
Model 2	Age ≥ 80 years	7.232	23.485	−41.351 to 55.815	0.308	0.761	0.334	0.009
	Body weight ≤ 60 kg	2.070	23.286	−46.102 to 50.241	0.089	0.930		
	Creatinine ≥ 1.5 mg/dL	72.543	24.987	19.855 to 123.232	2.863	0.009		
	Heart failure	47.446	23.212	−0.571 to 95.463	2.044	0.053

**Table 4:** Linear regression (multivariate analysis) for plasma apixaban concentration in all patients

		Estimated (β)	Standard error	95% CI	t-value	*p*-value	Adjusted *R*^2^	*p*-value
Model 3	Age ≥ 80 years	19.949	23.400	−27.211 to 67.110	0.853	0.399	0.183	0.011
	Body weight ≤ 60 kg	7.881	21.940	−36.336 to 52.098	0.359	0.721		
	Creatinine ≥ 1.5 mg/dL	65.710	26.474	12.355 to 119.065	2.482	0.017		
	Total dose of apixaban	16.153	4.611	6.861 to 22.445	3.503	0.001		
Model 4	Age ≥ 80 years	20.742	22.988	−25.617 to 67.101	0.902	0.372	0.212	0.009
	Body weight ≤ 60 kg	11.015	21.635	−32.616 to 54.647	0.509	0.613		
	Creatinine ≥ 1.5 mg/dL	59.245	26.307	6.192 to 112.298	2.252	0.029		
	Total dose of apixaban	16.268	4.529	7.135 to 25.402	3.592	< 0.001		
	Heart failure	32.515	20.107	−8.035 to 73.066	1.617	0.113		

**Fig. 2:**
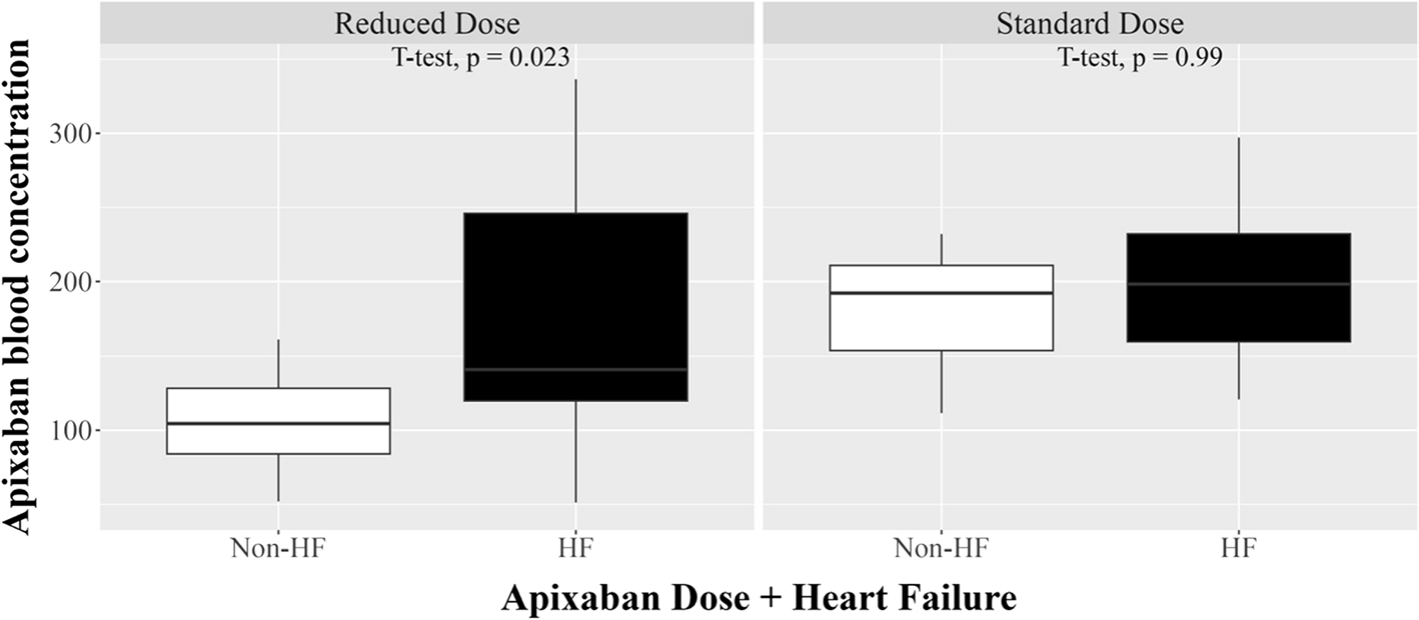
Plasma apixaban concentration based on heart failure and apixaban dose

When evaluating PAC by HF status, patients with dose reduction criteria ≥ 2 exhibited higher PAC depending on the presence of HF (Fig. 1D). Among those receiving reduced doses, the PAC was significantly higher in those with HF and comparable with that observed in patients receiving the standard dose, irrespective of HF (Fig. 2 and Supplemental Table 1).

### Bleeding events and plasma concentrations of apixaban

Among all patients, one case of major ISTH bleeding and four cases of CRNMB were observed (Supplementary Tables 2 and 3). All bleeding events occurred in the reduced-dose group. No significant differences were observed in variables other than HF when comparing patient characteristics and PAC based on bleeding events. Moreover, PAC showed no significant difference based on bleeding events (159.3 ± 75.5 vs. 169.2 ± 103.3 ng/mL, *p* = 0.791, Table 5). Stratification by dose and the presence of HF revealed a significant difference in plasma concentrations in the reduced-dose group, with higher bleeding rates observed in patients with HF taking a reduced dose (Table 6).

**Table 5:** Clinical characteristics and apixaban concentration based on bleeding

	Total (*N* = 49)	Bleeding (-) (*N* = 44)	Bleeding (+) (*N* = 5)	*p*-value
Age (year)	74.2 ± 7.7	73.7 ± 7.6	79.2 ± 7.2	0.127
Male	27 (55.1%)	23 (52.3%)	4 (80.0%)	0.480
Height (cm)	161.4 ± 8.2	161.4 ± 8.3	161.0 ± 7.7	0.902
Weight (kg)	63.5 ± 11.4	64.3 ± 11.4	56.9 ± 10.3	0.170
Hypertension	22 (44.9%)	22 (50.0%)	0 (0.0%)	0.098
Diabetes mellitus	18 (36.7%)	17 (38.6%)	1 (20.0%)	0.742
Dyslipidemia	8 (16.3%)	7 (15.9%)	1 (20.0%)	1.000
Liver disease ^†^	9 (18.4%)	7 (15.9%)	2 (40.0%)	0.478
Lung disease ^††^	14 (28.6%)	12 (27.3%)	2 (40.0%)	0.941
Chronic kidney disease ^†††^	23 (46.9%)	20 (45.5%)	3 (60.0%)	0.885
Creatinine (mg/dL)	1.3 ± 0.9	1.2 ± 1.0	1.7 ± 0.7	0.322
Creatinine clearance (mL/min)	54.0 ± 23.5	56.1 ± 22.8	35.2 ± 23.3	0.058
Heart failure	22 (44.9%)	17 (38.6%)	5 (100.0%)	0.032
Ischemic heart disease	8 (16.3%)	8 (18.2%)	0 (0.0%)	0.686
Stroke	9 (18.4%)	8 (18.2%)	1 (20.0%)	1.000
Malignancy	9 (18.8%)	7 (16.3%)	2 (40.0%)	0.496
Paroxysmal atrial fibrillation	6 (12.2%)	6 (13.6%)	0 (0.0%)	0.872
CHA_2_DS_2_-VAS score	3.4 ± 1.5	3.5 ± 1.5	3.0 ± 0.7	0.502
Aspirin	4 (8.2%)	4 (9.1%)	0 (0.0%)	1.000
Clopidogrel	2 (4.1%)	2 (4.5%)	0 (0.0%)	1.000
Apixaban concentration (ng/mL)	160.3 ± 77.5	159.3 ± 75.5	169.2 ± 103.3	0.791

† Liver diseases included cirrhosis and fatty liver

†† Lung diseases included chronic obstructive lung disease, asthma, and pulmonary tuberculosis

††† Chronic kidney disease was defined as an estimated glomerular filtration rate (eGFR) of 60 ml/min/1.73 m2s

**Table 6:** Outcomes and dose adjustment factors based on heart failure and apixaban dose

	Apixaban 2.5 mg twice daily			Apixaban 5 mg twice daily		
	Non-HF (*N* = 15)	HF (*N* = 13)	*p*-value	Non-HF (*N* = 12)	HF (*N* = 9)	*p*-value
Bleeding^a^	0 (0.0%)	5 (38.5%)	0.031	0 (0.0%)	0 (0.0%)	1.000
Apixaban concentration (ng/mL)	106.5 ± 31.7	172.6 ± 89.2	0.023	191.5 ± 79.3	190.8 ± 74.4	0.985
Age ≥ 80 years	7 (46.7%)	6 (46.2%)	1.000	2 (16.7%)	1 (11.1%)	1.000
Body Weight ≤ 60 kg	8 (53.3%)	7 (53.8%)	1.000	4 (33.3%)	1 (11.1%)	0.506
Serum creatinine ≥ 1.5 mg/dL	3 (20.0%)	6 (46.2%)	0.284	1 (8.3%)	0 (0.0%)	1.000
Creatinine clearance (mL/min)	52.2 ± 23.5	33.8 ± 17.2	0.028	59.1 ± 10.4	79.2 ± 18.2	0.005

^a^Composite of ISTH major bleeding and CRNMB

## Discussion

In this study, PAC was assessed using blood samples collected during routine laboratory tests, followed by detailed analysis. Notably, the factors influencing drug concentration differed between the standard- and reduced-dose groups. In the reduced-dose group, patients with HF exhibited a higher PAC than those without, which was associated with a higher incidence of bleeding events.

A study on the pharmacokinetics and pharmacodynamics of apixaban in healthy individuals revealed that the PAC in those receiving 2.5 mg twice daily was approximately 50% lower than that in those receiving 5.0 mg twice daily [9]. Another study on apixaban levels in real-world patients, along with findings from the AVERROES sub-study, showed that the PAC in patients receiving a reduced dose was approximately 20% lower than that in patients receiving the standard dose [10, 11]. Furthermore, in the present study, patients who received 2.5 mg of apixaban twice daily showed a PAC that was 30% lower than that observed with the standard dose. However, among patients receiving the reduced dose, the PAC in those without HF was approximately half the standard dose. In contrast, the PAC in patients with HF reached approximately 90% of the standard dose.

PAC can be influenced by factors such as ethnicity, sex, and comorbidities [6, 11, 12]. However, the use of apixaban is guided by specific dosing criteria. For patients with a creatinine clearance of ≥ 30 mL/min, if two or more of the following factors apply—age ≥ 80 years, body weight ≤ 60 kg, and serum creatinine ≥ 1.5 mg/dL—apixaban 2.5 mg twice daily was prescribed. For patients with a creatinine clearance rate of < 30 mL/min, apixaban 2.5 mg was administered twice daily [3]. However, blood pressure and blood flow can affect renal function, which may worsen HF, potentially resulting in reduced renal function [13]. Angiotensin-converting enzyme inhibitors and diuretics, which are mostly used in patients with HF, have a modest predictive value for worsening renal impairment [14]. Although the dose reduction criteria were implemented using a creatinine level of 1.5 mg/dL, older and underweight patients may still exhibit fluctuations in kidney function, which the presence of HF can influence. This may have affected the PAC.

In a previous study, patients receiving a reduced dose of apixaban had baseline characteristics that differed from those of patients receiving a standard dose. They were older, weighed less, and had decreased renal function compared with those in the standard-dose group. In addition, the reduced-dose group had a higher incidence of hemorrhage [15–19]. Despite the small sample size, the present study yielded similar results. However, whether HF directly causes bleeding remains unclear. In a subanalysis of the ARISTOTLE study, a left ventricular systolic dysfunction ≤ 40% was not associated with bleeding in patients taking apixaban [20]. In contrast, in the AFFIRM study, HF was associated with a higher risk of hemorrhage and stroke in patients with AF taking warfarin [21]. In the STANDARD study, which showed a higher risk of bleeding in the reduced-dose group, the proportion of patients with HF was significantly higher [15]. Patients with HF frequently have comorbidities that predict an increased risk of bleeding, including old age and renal and liver diseases [22]. Moreover, individuals who received a reduced dose of apixaban showed a higher prevalence of these conditions than those who received a standard dose.

**Fig. 3:**
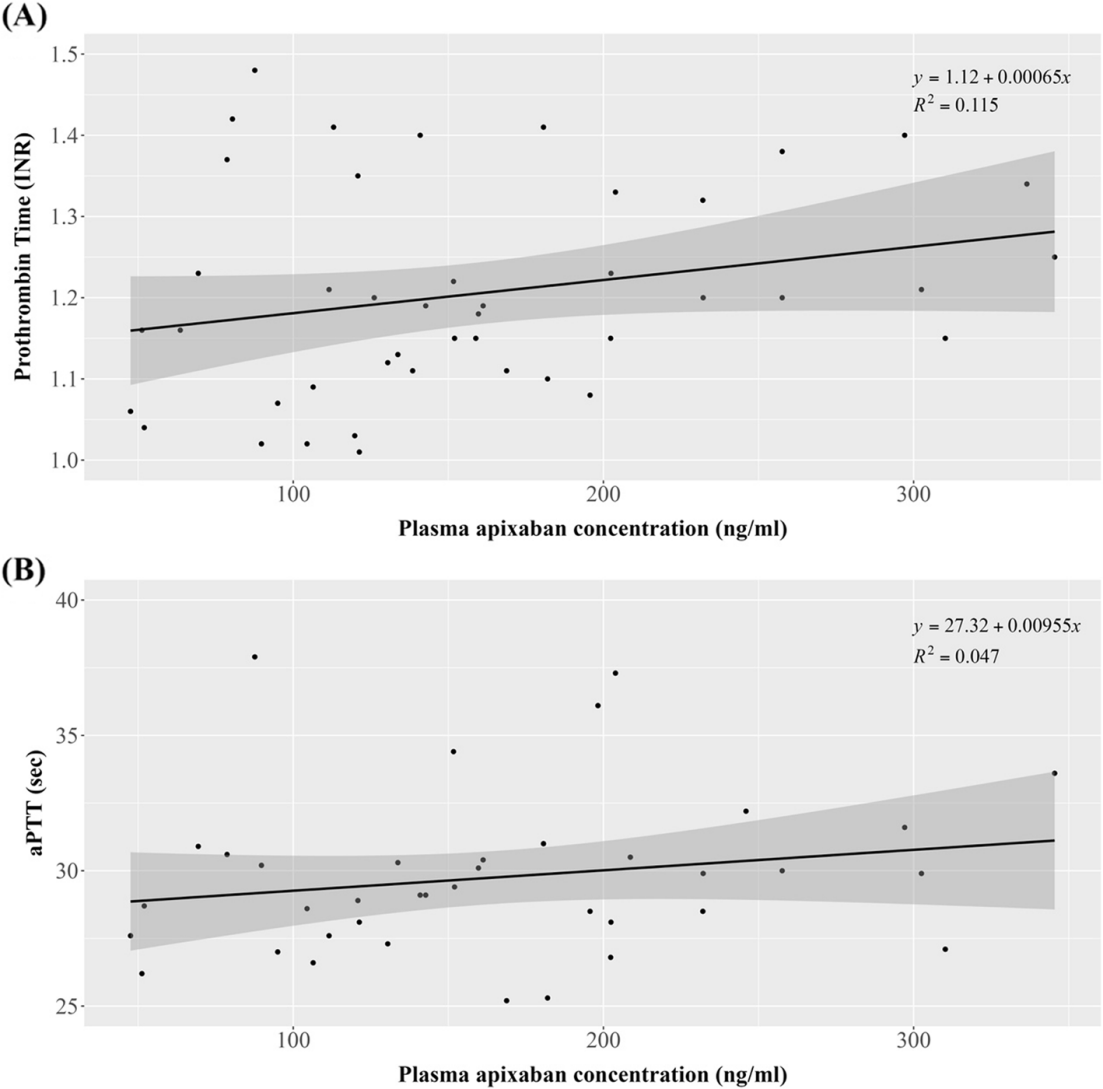
Relationship between plasma apixaban concentration and coagulation parameters. **A** Prothrombin time (INR) and **B** activated partial thromboplastin time (aPTT) are plotted against the plasma apixaban concentration (ng/mL). Black lines indicate linear regression with 95% confidence intervals

Although routine coagulation parameters such as PT and aPTT are commonly available, their utility in monitoring apixaban levels is limited. In our analysis, the PAC showed only a weak positive correlation with PT (INR) and aPTT (*R*^2^ = 0.115 and 0.047, respectively), and the regression slopes indicated modest increases in these parameters with increasing apixaban levels. These findings underscore the limited sensitivity of PT and aPTT in estimating apixaban exposure, suggesting that they are unreliable surrogates for plasma concentration monitoring (Fig. 3).

This study has limitations. First, this was a single-center, small-scale registry study. Second, the exact peak apixaban level could not be assessed because of variations in the timing of sample collection. Blood samples were obtained during outpatient visits or hospital stays, and the timing of specimen collection was not standardized. Consequently, the peak apixaban levels could not be measured, and discrepancies in drug concentrations resulting from variations in sample collection timing may have influenced the findings.

## Conclusion

Routine measurements of PAC showed that most patients receiving apixaban had appropriate doses determined by known age, body weight, and estimated GFR. However, HF may increase plasma concentrations and be associated with a higher risk of bleeding in Korean patients receiving a reduced dose. This finding suggests that individualized consideration of HF and other comorbidities plays a role in optimizing the prescription of reduced-dose apixaban.

## Supplementary Information

Supplementary Material 1.

## Data Availability

No datasets were generated or analysed during the current study.
