# Supplementary material for: Real-world apixaban concentration in Korean patients with atrial fibrillation
Source: Blood Res. 2025 Jul 7;60(1):39. doi: 10.1007/s44313-025-00089-z (PMC12234948; doi:10.1007/s44313-025-00089-z)
Supplement: Supplementary file 1 — Supplementary Material 1. [file 44313_2025_89_MOESM1_ESM.docx]

Supplement Table 1. Baseline characteristics based on apixaban dose and heart failure.

|  | Total (N=49) | Apixaban 2.5 mg twice daily | | | | Apixaban 5 mg twice daily | | | |
| --- | --- | --- | --- | --- | --- | --- | --- | --- | --- |
|  |  | Total  N=28 | Non-HF  (N=15) | HF  (N=13) | P-value | Total  N=21 | Non-HF  (N=12) | HF  (N=9) | P-value |
| Age (year) | 74.2 ± 7.7 | 77.4 ± 6.7 | 77.1 ± 7.4 | 77.7 ± 6.2 | 0.811 | 70.0 ± 6.9 | 71.5 ± 6.4 | 68.1 ± 7.5 | 0.278 |
| Male | 27 (55.1%) | 15 (53.6%) | 7 (46.7%) | 8 (61.5%) | 0.684 | 12 (57.1%) | 6 (50.0%) | 6 (66.7%) | 0.750 |
| Height (cm) | 161.4 ± 8.2 | 159.5 ± 7.9 | 159.4 ± 6.8 | 159.5 ± 9.3 | 0.983 | 164.0 ± 8.0 | 161.8 ± 7.2 | 166.8 ± 8.5 | 0.161 |
| Weight (kg) | 63.5 ± 11.4 | 58.6 ± 10.5 | 60.6 ± 11.2 | 56.2 ± 9.6 | 0.270 | 70.2 ± 9.0 | 69.0 ± 9.0 | 71.7 ± 9.4 | 0.510 |
| Hypertension | 22 (44.9%) | 13 (46.4%) | 8 (53.3%) | 5 (38.5%) | 0.684 | 9 (42.9%) | 7 (58.3%) | 2 (22.2%) | 0.227 |
| Diabetes mellitus | 18 (36.7%) | 8 (28.6%) | 3 (20.0%) | 5 (38.5%) | 0.510 | 10 (47.6%) | 7 (58.3%) | 3 (33.3%) | 0.488 |
| Dyslipidemia | 8 (16.3%) | 2 (7.1%) | 0 (0.0%) | 2 (15.4%) | 0.400 | 6 (28.6%) | 5 (41.7%) | 1 (11.1%) | 0.296 |
| Liver disease ^†^ | 9 (18.4%) | 7 (25.0%) | 5 (33.3%) | 2 (15.4%) | 0.512 | 2 (9.5%) | 0 (0.0%) | 2 (22.2%) | 0.334 |
| Lung disease ^††^ | 14 (28.6%) | 10 (35.7%) | 3 (20.0%) | 7 (53.8%) | 0.142 | 4 (19.0%) | 2 (16.7%) | 2 (22.2%) | 1.000 |
| Chronic kidney disease ^†††^ | 23 (46.9%) | 15 (53.6%) | 6 (40.0%) | 9 (69.2%) | 0.243 | 8 (38.1%) | 6 (50.0%) | 2 (22.2%) | 0.399 |
| Ischemic heart disease | 8 (16.3%) | 5 (17.9%) | 3 (20.0%) | 2 (15.4%) | 1.000 | 3 (14.3%) | 3 (25.0%) | 0 (0.0%) | 0.322 |
| Stroke | 9 (18.4%) | 6 (21.4%) | 3 (20.0%) | 3 (23.1%) | 1.000 | 3 (14.3%) | 1 (8.3%) | 2 (22.2%) | 0.787 |
| Malignancy | 9 (18.8%) | 8 (29.6%) | 4 (28.6%) | 4 (30.8%) | 1.000 | 1 (4.8%) | 0 (0.0%) | 1 (11.1%) | 0.882 |
| CHA_2_DS_2_-VAS score | 3.4 ± 1.5 | 3.8 ± 1.4 | 3.6 ± 1.5 | 3.9 ± 1.4 | 0.561 | 3.0 ± 1.5 | 3.1 ± 1.4 | 2.9 ± 1.7 | 0.775 |

Supplement Table 2. Clinical and laboratory outcomes based on apixaban dose.

| Daily dose | Total (N=49) | 2.5mg twice daily (N=28) | 5.0mg twice daily (N=21) | P-value |
| --- | --- | --- | --- | --- |
| Apixaban concentration | 160.3 ± 77.5 | 137.2 ± 72.0 | 191.2 ± 75.3 | 0.014 |
| Prothrombin Time (sec) | 13.8 ± 1.6 | 13.7 ± 1.7 | 13.9 ± 1.6 | 0.651 |
| PT INR | 1.2 ± 0.1 | 1.2 ± 0.2 | 1.2 ± 0.1 | 0.649 |
| aPTT (sec) | 28.9 ± 3.5 | 28.8 ± 3.4 | 28.9 ± 3.7 | 0.915 |
| Bleeding | 5 (10.2%) | 5 (17.9%) | 0 (0.0%) | 0.117 |
| ISTH major bleeding | 1 (2.0%) | 1 (3.6%) | 0 (0.0%) | 1.000 |
| CRNMB | 4 (8.2%) | 4 (14.3%) | 0 (0.0%) | 0.200 |

･ ISTH: the International Society on Thrombosis and Haemostasis

･ CRNMB: clinically relevant non-major bleeding

Supplement Table 3. List of patients with bleeding event.

| Age | Sex | Body weight (kg) | Creatinine (mg/dL) | Apixaban concentration (ng/ml) | Duration (day) | Bleeding | Type |
| --- | --- | --- | --- | --- | --- | --- | --- |
| 86 | M | 55 | 1.8 | 63.6 | 30 | subdural hemorrhage | major bleeding |
| 70 | M | 71 | 1.0 | 113.0 | 90 | hematuria | CRNMB |
| 80 | M | 55 | 1.0 | 152.0 | 89 | hematochezia | CRNMB |
| 74 | M | 60 | 2.8 | 180.7 | 222 | melena | CRNMB |
| 86 | F | 42 | 1.8 | 336.5 | 120 | melena | CRNMB |

･ ISTH: the International Society on Thrombosis and Haemostasis

･ CRNMB: clinically relevant non-major bleeding
